# Treatment of Juvenile Idiopathic Arthritis-Associated Uveitis

**DOI:** 10.4274/tjo.09581

**Published:** 2016-04-05

**Authors:** Merih Oray, İlknur Tuğal-Tutkun

**Affiliations:** 1 İstanbul University İstanbul Faculty of Medicine, Department of Ophthalmology, İstanbul, Turkey

**Keywords:** Uveitis, juvenile idiopathic arthritis, antimetabolites, biologic agents

## Abstract

Pediatric uveitis may be a serious health problem because of the lifetime burden of vision loss due to severe complications if the problem is not adequately treated. Juvenile idiopathic arthritis (JIA)-associated uveitis is characterized by insidious onset and potentially blinding chronic anterior uveitis. Periodic ophthalmologic screening is of utmost importance for early diagnosis of uveitis. Early diagnosis and proper immunomodulatory treatment are essential for good visual prognosis. The goal of treatment is to achieve enduring drug-free remission. The choice of therapeutic regimen needs to be tailored to each individual case. One must keep in mind that patients under immunomodulatory treatment should be monitored closely due to possible side effects. Local and systemic corticosteroids have long been the mainstay of therapy; however, long-term corticosteroid therapy should be avoided due to serious side effects. Steroid-sparing agents in the treatment of JIA-associated uveitis include antimetabolites and biologic agents in refractory cases. Among the various immunomodulatory agents, methotrexate is generally the first choice, as it has a well-established safety and efficacy profile in pediatric cases and does not appear to increase the risk of cancer. Other classic immunomodulators that may also be used in combination with methotrexate include azathioprine, mycophenolate mofetil, and cyclosporin A. Biologic agents, primarily tumor necrosis factor alpha inhibitors including infliximab or adalimumab, should be considered in cases of treatment failure with classic immunomodulatory agents.

## INTRODUCTION

Despite advances in diagnosis and treatment, uveitis, especially in the pediatric age group, continues to be a serious health problem due to complications that may lead to blindness. Ocular involvement has particular importance in extra-articular manifestations of pediatric rheumatic diseases because of its high incidence and morbidity. Juvenile idiopathic arthritis (JIA) is the most common pediatric rheumatic disease with both articular and ocular involvement.^1,2,3,4,5^

In the United States of America, 6% of all reported uveitis cases are pediatric, and approximately 80% of these are related to JIA.^6,7^ In Turkey, JIA and Behçet’s disease are the most common systemic diseases among pediatric uveitis cases, and the reported incidence of JIA varies between 3.3% and 30.4%.^8,9,10,11^

JIA is characterized by chronic arthritis beginning before the age of 16 and is the leading cause of arthritis in pediatric patients. It occurs more frequently in female children, with a reported female to male ratio of 3:2. The International League of Associations of Rheumatology (ILAR) classification system defines 7 subtypes of JIA which feature varying rates and types of uveitis. Approximately 78-90% of patients with JIA-associated uveitis have oligoarticular (≤4 joints) manifestation and 90% of these patients are antinuclear antibody (ANA) positive. Between 7-14% of the patients have polyarticular (≥5 joints) and 2-6% have systemic (systemic symptoms as well as articular involvement) manifestations. The average age of uveitis onset in JIA patients is 6-8 years old. In the majority of patients uveitis appears within 4-7 years of arthritis onset. However, uveitis occurs prior to arthritis in about 6% of cases and is only noticed if an eye exam is performed when the arthritis is diagnosed.^12,13,14,15,16^ Therefore, it is imperative that all patients diagnosed with JIA undergo ophthalmologic examination and regular screening depending on the disease type. Oligoarticular and polyarticular JIA patients with arthritis onset at or before age 6, with arthritis for 4 years or less or positive for ANA should undergo an ophthalmologic examination every 3 months. Screening intervals for patients at lower risk of uveitis should be 6 to 12 months.17 The diagnosis may be overlooked due to a lack of obvious ocular symptoms like redness, pain or light sensitivity, because some pediatric patients are unable to sufficiently communicate, or due to the chronic course of the disease. As a result, serious sight-threatening complications such as band keratopathy, cataract, glaucoma or hypotony may be observed at presentation.^1,18,19,20^

Patients with a consistently high degree of flare in the aqueous humour, which indicates the protein level, are at greater risk of complications. Risk factors for a poor prognosis are early age of uveitis onset, male gender, ANA positivity, short interval between arthritis and uveitis onset, oligoarticular manifestation and presence of ocular complications at time of presentation. Furthermore, patients with onset of arthritic involvement in early childhood are at high risk of chronic severe uveitis. In contrast, patients with arthritis onset at a later age exhibit recurrent acute anterior uveitis attacks and have a better prognosis.^18,21^ The early diagnosis and correct treatment of these pediatric patients is critical for a good visual prognosis.

Cases of JIA-associated uveitis typically exhibit anterior uveitis characterized by iris and ciliary body involvement and is often bilateral. As a patient’s arthritis and uveitis may follow different courses, the activity in each area of involvement should be evaluated independently, and treatment should be planned for each individual in cooperation with a pediatric rheumatologist. The results of numerous studies in the literature related to this topic can be used as the basis for a specific treatment algorithm for pediatric uveitis patients.^6,7,22^

## CORTICOSTEROIDS

Corticosteroids are essential in the treatment of uveitis, but their prolonged use is discouraged in order to avoid serious side effects, particularly in pediatric patients. Besides corticosteroids, long-term treatment options for the treatment of JIA-associated uveitis include antimetabolites, alkylating agents and biologic agents. These agents may also lead to serious side effects, and their risks to pediatric patients must be thoroughly evaluated; patients should be followed closely together with pediatric rheumatologists using a multidisciplinary approach.^6,7,23,24^

Topical corticosteroids and mydriatic agents can be administered as first-line treatment for patients with mild anterior uveitis diagnosed prior to the development of ocular complications (early band keratopathy, posterior synechia, cataract, macular edema). Prednisolone acetate (Predforte®), dexamethasone (Maxidex®, Dexasine®), fluorometholone (Flarex®, FML®) and loteprednol etabonate (Lotemax®) are the ophthalmic corticosteroids used in Turkey. Among these, prednisolone acetate and dexamethasone are the most potent and are the first choice in our clinical practice for the treatment of uveitis. Frequency of topical corticosteroid use should be evaluated for each patient based on inflammation severity. It is important to monitor intraocular pressure during the use of topical corticosteroids, especially in pediatric patients. Patients should be followed closely; if satisfactory treatment response is observed, the number of topical corticosteroid drops applied can be gradually decreased. However, in cases of insufficient treatment response or recurrence when the topical dose is reduced, local corticosteroid injections can be administered or short-term systemic corticosteroids can be added to the treatment regimen. Elevated intraocular pressure commonly occurs following intravitreal, peribulbar or sub-Tenon’s corticosteroid (triamcinolone acetonide) injections, especially in pediatric patients. The recently introduced intravitreal dexamethasone implant (Ozurdex®, Allergan Inc, Irvine, CA, USA) has not caused significant intraocular pressure elevation in the majority of pediatric patients, and therefore may be preferable for cases requiring local corticosteroid injections.^25,26^ However, it should be kept in mind that these implants may also lead to serious intraocular pressure elevation in some patients. Therefore, careful patient selection is important.^27^

As systemic therapy, 1-2 mg/kg oral prednisolone (Deltacortril® tablet) or methylprednisolone (Prednol® tablet) can be initiated as a loading dose, whereas 30 mg/kg intravenous pulse methylprednisolone (Prednol® ampoule) can be administered if a more rapid and potent effect is desired. Just as long-term topical corticosteroid use or local steroid injection can lead to ocular side effects like cataract and glaucoma, long-term systemic corticosteroid use can cause serious side effects such as growth and developmental delays due to adrenal suppression and premature epiphyseal closure, weight loss, hyperglycemia, infection and osteoporosis.^28^ If a patient without ocular complications at the time of presentation exhibits recurrence when corticosteroid therapy is reduced, or elevated intraocular pressure is observed as a result of corticosteroid administration, immunomodulatory therapy should be started as soon as possible. However, patients presenting with at least one ocular complication in at least one eye require both systemic corticosteroid treatment and immunomodulatory therapy. Another point to be aware of is that although oral nonsteroid anti-inflammatory agents are effective for articular involvement in JIA patients, they have no effect in the treatment of uveitis.^29^

## ANTIMETABOLITES, T-CELL INHIBITORS AND ALKYLATING AGENTS

Methotrexate is the first choice in immunomodulatory therapy because its efficacy and safety in pediatric patients is well established, and its long-term use does not increase cancer risk.^1,6,7,12,13,30,31^ Approximately 60-82% of JIA-associated uveitis patients show improvement with methotrexate therapy.^1,6,31,32^ Treatment with methotrexate is initiated early and continues for at least 3 years; a period of inactivity 2 years or longer before discontinuation lowers the risk for recurrence.^32^ Methotrexate therapy begins at 10-15 mg/m2 orally once per week and can be increased weekly for 6-8 weeks depending on response; a dose of up to 30 mg/m2/week can be tolerated safely.^7,33^ In JIA patients, the dose required for uveitis remission is generally higher than the dose administered for arthritis. In addition, as the dosage is set according to the patient’s mass, children’s weight should be followed regularly during growth periods. In cases where oral treatment is not tolerated by the patient or is not controlling the uveitis, treatment can be changed to a parenteral route, which provides better bioavailability. The potential side effects of methotrexate therapy include bone marrow suppression, hepatotoxicity and interstitial pneumonia. Because methotrexate is a folic acid analogue, folic acid should be added to treatment in order to prevent side effects.^31^ Aversion is another undesired side effect that can arise during methotrexate therapy. Prior to receiving an oral or injected dose, children frequently experience stomachache, nausea and may even vomit, symptoms which significantly impact the child’s quality of life. In such cases it is important not to insist on treatment and instead employ alternative agents.

JIA-associated uveitis can cause intractable inflammation.^6^ Samson et al.^34^ found that uveitis symptoms were controlled with methotrexate in 59% of 21 patients with recurrent or chronic JIA-associated uveitis, while 41% had persistent inflammation. Other immunomodulatory agents or combined therapy can be started in cases where methotrexate is ineffective. Azathioprine, mycophenolate mofetil and cyclosporin are classic immunomodulatory agents alternative to methotrexate. Azathioprine is an effective agent for both adults and children, but is not preferred for pediatric patients due to its gastrointestinal side effects.^35,36^ Mycophenolate mofetil, a better tolerated antimetabolite agent, inhibits purine synthesis and can be administered as an alternative treatment in patients who are resistant to methotrexate.^37^ Cyclosporin A, a calcineurin inhibitor which interferes with T-cell activation, has limited efficacy in JIA-associated uveitis when used in isolation.^6^ It is often administered in combination with methotrexate.^38^ Cyclosporine can be used at 3-5 mg/kg/day.^39^ Other less frequently employed T-cell inhibitors are tacrolimus and sirolimus. However, there is insufficient data regarding the use of these agents in pediatric patients. These agents may also cause hypertension and nephrotoxicity; therefore patients should be followed closely.^7^

Alkylating agents are utilized in cases showing insufficient treatment response to antimetabolites or combination therapy.^6,30^ Although cyclophosphamide and chlorambucil are effective at suppressing inflammation, they have serious potential side effects such as pancytopenia, malignancy development in the long term, gonadal dysfunction and infertility.^40,41^ With the introduction of biologic agents in the treatment of ocular inflammation, the use of alkylating agents in pediatric patients is becoming less common.

## BIOLOGIC AGENTS

Among the monoclonal antibody biologic agents that suppress inflammation by binding proinflammatory cytokines, the most effective for ocular inflammation are the tumor necrosis factor (TNF)-alpha inhibitors infliximab and adalimumab. They can be used alone or in combination with classic immunomodulatory therapy.^7^

Infliximab is a chimeric mouse/human monoclonal antibody administered as an intravenous infusion at 5-20 mg/kg for 2 weeks as a loading dose and once every 4 weeks thereafter.^42^ Low-dose antimetabolite therapy is often administered concomitantly to prevent antichimeric antibody production.^43^ In our clinic we achieved successful outcomes in the short term with infliximab treatment in 20 pediatric patients with uveitis refractory to conventional treatment. Furthermore, successful surgical outcomes in cases with serious complications such as glaucoma and cataract have been achieved as a result of the anti-inflammatory effect of preoperative infliximab therapy. However, in long-term follow-up, 4 patients treated for 10-36 months reportedly developed resistance to treatment.^44^

Adalimumab, a fully human monoclonal antibody, is administered as a 20-40 mg subcutaneous injection every 7-14 days. In two separate clinical studies, Vazquez-Cobian et al.^45^ and Biester et al.^46^ demonstrated that adalimumab treatment was effective in 80.8% and 88% of pediatric uveitis cases, respectively. Although infliximab has a faster initial effect, evidence indicates that adalimumab is not associated with risk of inducing severe allergic reactions like anaphylaxis. Some studies comparing the efficacy of adalimumab and infliximab have shown that adalimumab is slightly more effective at inducing remission, while others have reported no significant differences between the two treatments.^47,48^ A clinical study comparing methotrexate/adalimumab combined treatment and adalimumab monotherapy is still in progress.^49^

Etanercept, another anti-TNF agent, has been determined effective in the treatment of other rheumatologic manifestations of JIA but has not shown sufficient efficacy in JIA-associated uveitis and is even reported to lead to relapses causing the emergence of uveitis.^50,51,52,53 ^Our knowledge concerning the efficacy of golimumab and certolizumab pegol, also in this group of biologics, is limited to case studies; randomized clinical studies investigating the efficacy of these agents have yet to be conducted.^54,55^

Other biologic agents targeting immune cells can be utilized with pediatric uveitis patients who do not respond to anti-TNF therapy. These include tocilizumab, an inhibitor of pro-inflammatory cytokine interleukin (IL)-6; rituximab, an anti-CD-20 monoclonal antibody; anakinra, an IL-1 antagonist; daclizumab, an IL-2 antagonist; and abatacept, a cytotoxic T lymphocyte-associated antigen 4 fusion protein that inhibits T cell co-stimulation. Some studies with small case numbers have demonstrated the efficacy of these agents in suppressing inflammation in refractory JIA-associated uveitis.^22,56,57,58,59,60^

## SURGICAL TREATMENT

Surgical intervention may be necessary in addition to medical treatment in cases of JIA-associated pediatric uveitis that develop complications like band keratopathy, cataract and glaucoma during follow-up. An important note to be aware of in these cases is that surgical success is highly dependent on the complete suppression of the ocular inflammation with medical treatment.^23^

## BAND KERATOPATHY

Band keratopathy is characterized by the deposition of calcium between the corneal epithelium and Bowman’s layer. It frequently originates in the limbus near the 3 and 9 o’clock positions. It is one of the sight-threatening complications of JIA-associated uveitis. In a study with a large case series of 327 patients, 34.1% were shown to have band keratopathy at diagnosis.^21^ When the condition threatens sight, chelation therapy with ethylenediaminetetraacetic acid (EDTA) can be performed. In a study by Najjar et al.^61^ reporting the long-term outcomes of EDTA chelation in the treatment of calcific band keratopathy, they demonstrated that the method is effective, but that uveitic eyes exhibit a high recurrence rate. Therefore, its application is recommended in eyes with severely threatened sight or amblyopia risk.

## CATARACT SURGERY

Cataract surgery in pediatric patients may be a challenge due to low scleral rigidity and existing ocular complications such as band keratopathy and posterior synechiae. In recent years, excellent visual outcomes have been achieved with good management of perioperative inflammation as well as modern surgical techniques and instruments. Intraocular lens (IOL) implantation during cataract surgery has been a controversy for many years. Until recently, the consensus was that IOL implantation was contraindicated in JIA-associated uveitis patients due to the severe postoperative inflammatory membrane formation around the IOL.62,63 However, data from more recent studies contradict this stance.^64,65^ Sijssens et al.^64^ detected no significant difference in the postoperative complication rates of 19 aphakic eyes and 29 pseudophakic eyes, and demonstrated that pseudophakic eyes maintained better visual acuity up to 7 years postoperatively.

## GLAUCOMA SURGERY

Glaucoma may occur in uveitic eyes as a result of various mechanisms including aqueous outflow blockage due to peripheral anterior synechiae, reduced aqueous outflow due to increased aqueous protein concentration, trabecular inflammation and secondary damage.^66,67^ Surgical intervention may be necessary in cases of failed medical treatment. The effectiveness of trabeculectomy is particularly limited in pediatric uveitis patients due to severe postoperative inflammation and fibrosis.^67,68,69^ Glaucoma drainage implant surgery and goniotomy are other surgical methods that may be employed in pediatric uveitis patients.^70,71,72,73,74^ An important point to keep in mind is that preoperative inflammation control is a crucial factor in the success of glaucoma surgery.

## CONCLUSIONS AND RECOMMENDATIONS

JIA-associated uveitis comprises a difficult disease group in terms of diagnosis and treatment. Diagnosing the condition before complications arise and starting immunomodulatory therapy immediately are critical for a good visual prognosis. Although the arthritis findings of patients with oligoarticular JIA may be inactive when uveitis is detected, treatment in that period should be planned according to uveitis activity. Appropriate treatment approaches can be planned on a case-by-case basis in order to achieve the ultimate aim of treatment, enduring remission after discontinuation of medication. The ocular complications which may occur as a result of disease-related inflammation and applied treatment methods as well as possible systemic complications resulting from immunomodulatory therapy should be monitored closely and treated immediately when necessary.

## Ethics

Peer-review: Externally peer-reviewed.
